# Supplementary material for: Overlapping cell population expression profiling and regulatory inference in C. elegans
Source: BMC Genomics. 2016 Feb 29;17:159. doi: 10.1186/s12864-016-2482-z (PMC4772325; doi:10.1186/s12864-016-2482-z)
Supplement: Additional file 13: — Web supplement. (DOC 21 kb) [file 12864_2016_2482_MOESM13_ESM.zip › sortWeb/clusters/hier.300.clusters/3.html]

Cluster 3 

## Cluster 3

### Expression

| cnd-1 rep. 1 | cnd-1 rep. 2 | cnd-1 rep. 3 | pha-4 rep. 1 | pha-4 rep. 2 | pha-4 rep. 3 | ceh-27 | ceh-36 | ceh-6 | F21D5.9 | mir-57 | mls-2 | pal-1 | pros-1 | ttx-3 | unc-130 | hlh-16 | irx-1 | ceh-6 (+) hlh-16 (+) | ceh-6 (+) hlh-16 (-) | ceh-6 (-) hlh-16 (+) | cnd-1 singlets | pha-4 singlets | 0 | 60 | 120 | 150 | 180 | 240 | 330 | 390 | 420 | 480 | 540 | 570 | 600 | 630 | 660 | NAME | Functional description |
| --- | --- | --- | --- | --- | --- | --- | --- | --- | --- | --- | --- | --- | --- | --- | --- | --- | --- | --- | --- | --- | --- | --- | --- | --- | --- | --- | --- | --- | --- | --- | --- | --- | --- | --- | --- | --- | --- | --- | --- |
|  |  |  |  |  |  |  |  |  |  |  |  |  |  |  |  |  |  |  |  |  |  |  |  |  |  |  |  |  |  |  |  |  |  |  |  |  |  | *pinn-4* | ParvulIN of Nematode |
|  |  |  |  |  |  |  |  |  |  |  |  |  |  |  |  |  |  |  |  |  |  |  |  |  |  |  |  |  |  |  |  |  |  |  |  |  |  | F23C8.5 |  |
|  |  |  |  |  |  |  |  |  |  |  |  |  |  |  |  |  |  |  |  |  |  |  |  |  |  |  |  |  |  |  |  |  |  |  |  |  |  | *isp-1* | Iron-Sulfur Protein |
|  |  |  |  |  |  |  |  |  |  |  |  |  |  |  |  |  |  |  |  |  |  |  |  |  |  |  |  |  |  |  |  |  |  |  |  |  |  | K05C4.2 |  |
|  |  |  |  |  |  |  |  |  |  |  |  |  |  |  |  |  |  |  |  |  |  |  |  |  |  |  |  |  |  |  |  |  |  |  |  |  |  | *ril-1* | RNAi-Induced Longevity |
|  |  |  |  |  |  |  |  |  |  |  |  |  |  |  |  |  |  |  |  |  |  |  |  |  |  |  |  |  |  |  |  |  |  |  |  |  |  | *cco-2* | Cytochrome C Oxidase |
|  |  |  |  |  |  |  |  |  |  |  |  |  |  |  |  |  |  |  |  |  |  |  |  |  |  |  |  |  |  |  |  |  |  |  |  |  |  | *ulp-3* | Ubiquitin-Like Protease |
|  |  |  |  |  |  |  |  |  |  |  |  |  |  |  |  |  |  |  |  |  |  |  |  |  |  |  |  |  |  |  |  |  |  |  |  |  |  | Y39A1A.3 |  |
|  |  |  |  |  |  |  |  |  |  |  |  |  |  |  |  |  |  |  |  |  |  |  |  |  |  |  |  |  |  |  |  |  |  |  |  |  |  | *rpc-11* | RNA Polymerase, Class III (C) |
|  |  |  |  |  |  |  |  |  |  |  |  |  |  |  |  |  |  |  |  |  |  |  |  |  |  |  |  |  |  |  |  |  |  |  |  |  |  | *rpl-24.2* | Ribosomal Protein, Large subunit |
|  |  |  |  |  |  |  |  |  |  |  |  |  |  |  |  |  |  |  |  |  |  |  |  |  |  |  |  |  |  |  |  |  |  |  |  |  |  | Y40B1B.7 |  |
|  |  |  |  |  |  |  |  |  |  |  |  |  |  |  |  |  |  |  |  |  |  |  |  |  |  |  |  |  |  |  |  |  |  |  |  |  |  | W09C5.8 |  |
|  |  |  |  |  |  |  |  |  |  |  |  |  |  |  |  |  |  |  |  |  |  |  |  |  |  |  |  |  |  |  |  |  |  |  |  |  |  | F58F12.1 |  |
|  |  |  |  |  |  |  |  |  |  |  |  |  |  |  |  |  |  |  |  |  |  |  |  |  |  |  |  |  |  |  |  |  |  |  |  |  |  | W08F4.11 |  |
|  |  |  |  |  |  |  |  |  |  |  |  |  |  |  |  |  |  |  |  |  |  |  |  |  |  |  |  |  |  |  |  |  |  |  |  |  |  | Y51H7C.7 |  |
|  |  |  |  |  |  |  |  |  |  |  |  |  |  |  |  |  |  |  |  |  |  |  |  |  |  |  |  |  |  |  |  |  |  |  |  |  |  | *tag-280* | Temporarily Assigned Gene name |
|  |  |  |  |  |  |  |  |  |  |  |  |  |  |  |  |  |  |  |  |  |  |  |  |  |  |  |  |  |  |  |  |  |  |  |  |  |  | T20D3.6 |  |
|  |  |  |  |  |  |  |  |  |  |  |  |  |  |  |  |  |  |  |  |  |  |  |  |  |  |  |  |  |  |  |  |  |  |  |  |  |  | *acbp-1* | Acyl-Coenzyme A Binding Protein |
|  |  |  |  |  |  |  |  |  |  |  |  |  |  |  |  |  |  |  |  |  |  |  |  |  |  |  |  |  |  |  |  |  |  |  |  |  |  | F44E5.1 |  |
|  |  |  |  |  |  |  |  |  |  |  |  |  |  |  |  |  |  |  |  |  |  |  |  |  |  |  |  |  |  |  |  |  |  |  |  |  |  | Y74C9A.6 |  |
|  |  |  |  |  |  |  |  |  |  |  |  |  |  |  |  |  |  |  |  |  |  |  |  |  |  |  |  |  |  |  |  |  |  |  |  |  |  | K11H12.1 |  |
|  |  |  |  |  |  |  |  |  |  |  |  |  |  |  |  |  |  |  |  |  |  |  |  |  |  |  |  |  |  |  |  |  |  |  |  |  |  | *asg-1* | ATP Synthase G homolog |
|  |  |  |  |  |  |  |  |  |  |  |  |  |  |  |  |  |  |  |  |  |  |  |  |  |  |  |  |  |  |  |  |  |  |  |  |  |  | Y53F4B.14 |  |
|  |  |  |  |  |  |  |  |  |  |  |  |  |  |  |  |  |  |  |  |  |  |  |  |  |  |  |  |  |  |  |  |  |  |  |  |  |  | *ife-2* | Initiation Factor 4E (eIF4E) family |
|  |  |  |  |  |  |  |  |  |  |  |  |  |  |  |  |  |  |  |  |  |  |  |  |  |  |  |  |  |  |  |  |  |  |  |  |  |  | Y66D12A.9 |  |
|  |  |  |  |  |  |  |  |  |  |  |  |  |  |  |  |  |  |  |  |  |  |  |  |  |  |  |  |  |  |  |  |  |  |  |  |  |  | F56B3.11 |  |
|  |  |  |  |  |  |  |  |  |  |  |  |  |  |  |  |  |  |  |  |  |  |  |  |  |  |  |  |  |  |  |  |  |  |  |  |  |  | C15C7.4 |  |
|  |  |  |  |  |  |  |  |  |  |  |  |  |  |  |  |  |  |  |  |  |  |  |  |  |  |  |  |  |  |  |  |  |  |  |  |  |  | F44E7.9 |  |
|  |  |  |  |  |  |  |  |  |  |  |  |  |  |  |  |  |  |  |  |  |  |  |  |  |  |  |  |  |  |  |  |  |  |  |  |  |  | *hrg-1* | Heme Responsive Gene |
|  |  |  |  |  |  |  |  |  |  |  |  |  |  |  |  |  |  |  |  |  |  |  |  |  |  |  |  |  |  |  |  |  |  |  |  |  |  | C34H4.2 |  |
|  |  |  |  |  |  |  |  |  |  |  |  |  |  |  |  |  |  |  |  |  |  |  |  |  |  |  |  |  |  |  |  |  |  |  |  |  |  | C49A9.4 |  |
|  |  |  |  |  |  |  |  |  |  |  |  |  |  |  |  |  |  |  |  |  |  |  |  |  |  |  |  |  |  |  |  |  |  |  |  |  |  | *lys-4* | LYSozyme |
|  |  |  |  |  |  |  |  |  |  |  |  |  |  |  |  |  |  |  |  |  |  |  |  |  |  |  |  |  |  |  |  |  |  |  |  |  |  | C24B9.3 |  |
|  |  |  |  |  |  |  |  |  |  |  |  |  |  |  |  |  |  |  |  |  |  |  |  |  |  |  |  |  |  |  |  |  |  |  |  |  |  | *ugt-17* | UDP-GlucuronosylTransferase |
|  |  |  |  |  |  |  |  |  |  |  |  |  |  |  |  |  |  |  |  |  |  |  |  |  |  |  |  |  |  |  |  |  |  |  |  |  |  | C25F9.6 |  |
|  |  |  |  |  |  |  |  |  |  |  |  |  |  |  |  |  |  |  |  |  |  |  |  |  |  |  |  |  |  |  |  |  |  |  |  |  |  | *mlc-4* | Myosin Light Chain |
|  |  |  |  |  |  |  |  |  |  |  |  |  |  |  |  |  |  |  |  |  |  |  |  |  |  |  |  |  |  |  |  |  |  |  |  |  |  | *his-7* | HIStone |
|  |  |  |  |  |  |  |  |  |  |  |  |  |  |  |  |  |  |  |  |  |  |  |  |  |  |  |  |  |  |  |  |  |  |  |  |  |  | Y23H5A.8 |  |
|  |  |  |  |  |  |  |  |  |  |  |  |  |  |  |  |  |  |  |  |  |  |  |  |  |  |  |  |  |  |  |  |  |  |  |  |  |  | *ndx-8* | NuDiX family |
|  |  |  |  |  |  |  |  |  |  |  |  |  |  |  |  |  |  |  |  |  |  |  |  |  |  |  |  |  |  |  |  |  |  |  |  |  |  | K08F8.11 |  |
|  |  |  |  |  |  |  |  |  |  |  |  |  |  |  |  |  |  |  |  |  |  |  |  |  |  |  |  |  |  |  |  |  |  |  |  |  |  | *klf-1* | Kruppel-Like Factor (zinc finger protein) |
|  |  |  |  |  |  |  |  |  |  |  |  |  |  |  |  |  |  |  |  |  |  |  |  |  |  |  |  |  |  |  |  |  |  |  |  |  |  | F41D9.11 |  |
|  |  |  |  |  |  |  |  |  |  |  |  |  |  |  |  |  |  |  |  |  |  |  |  |  |  |  |  |  |  |  |  |  |  |  |  |  |  | C45G3.4 |  |
|  |  |  |  |  |  |  |  |  |  |  |  |  |  |  |  |  |  |  |  |  |  |  |  |  |  |  |  |  |  |  |  |  |  |  |  |  |  | F32H5.3 |  |
|  |  |  |  |  |  |  |  |  |  |  |  |  |  |  |  |  |  |  |  |  |  |  |  |  |  |  |  |  |  |  |  |  |  |  |  |  |  | K01A2.9 |  |
|  |  |  |  |  |  |  |  |  |  |  |  |  |  |  |  |  |  |  |  |  |  |  |  |  |  |  |  |  |  |  |  |  |  |  |  |  |  | *sru-43* | Serpentine Receptor, class U |

### Phenotypes enriched

|  |  |  |  |
| --- | --- | --- | --- |
| **Group name** | **Number in cluster** | **Enrichment** | **FDR corrected p** |
| mitochondrial metabolism variant (RNAi) | 4 | 41.33 | 0.00395 |
| organelle metabolism variant (RNAi) | 4 | 32.47 | 0.00919 |

### Anatomy terms enriched

none found

### GO terms enriched

|  |  |  |
| --- | --- | --- |
| **GO term** | **Number of genes** | **FDR-corrected p-value** |
| intracellular organelle part | 7 | 0.024 |
| organelle membrane | 3 | 0.028 |
| mitochondrial inner membrane | 3 | 0.029 |
| cytoplasm | 9 | 0.039 |

### Expression clusters enriched

|  |  |  |  |
| --- | --- | --- | --- |
| **Group name** | **Number in cluster** | **Enrichment** | **FDR corrected p** |
| The cluster contains genes that are significantly enriched in L1 muscle. | 16 | 3.78 | 0.00063 |
| Genes in the top 10% of expression level across the triplicate L3 samples. To generate the top10 and bottom10 gene sets, authors ranked all genes by mean expression array signal intensity across the three replicates, then took the top and bottom deciles (1,841 genes each) to represent genes with high and low expression. | 17 | 3.15 | 0.00269 |
| Differentially expressed genes during worm lifespan. Medoid 7 Fig.4. | 7 | 7.19 | 0.01230 |
| monovalent inorganic cation transporter activity; 7.8e-06(54.75x). hydrogen ion transporter activity; 7.8e-06(54.75x). primary active transporter activity; 2.0e-05(41.06x). carrier activity; 9.1e-05(25.27x). cation transporter activity; 1.1e-04(22.66x). ion transporter activity; 2.3e-04(18.25x). energy pathways; 6.0e-04(13.69x). | 2 | 170.47 | 0.01300 |
| monovalent inorganic cation transporter activity; 1.9e-05(43.80x). hydrogen ion transporter activity; 1.9e-05(43.80x). energy pathways; 3.8e-05(14.60x). primary active transporter activity; 4.9e-05(32.85x). coenzymes and prosthetic group metabolism; 9.9e-05(26.28x). cofactor metabolism; 9.9e-05(26.28x). coenzyme metabolism; 9.9e-05(26.28x). carrier activity; 2.2e-04(20.22x). cation transporter activity; 2.8e-04(18.12x). ion transporter activity; 5.5e-04(14.60x). | 2 | 136.37 | 0.02050 |
| Genes enriched in intestine. | 16 | 2.72 | 0.02560 |
| energy pathways; 2.3e-06(15.21x). coenzymes and prosthetic group metabolism; 2.9e-06(29.20x). cofactor metabolism; 2.9e-06(29.20x). coenzyme metabolism; 2.9e-06(29.20x). monovalent inorganic cation transporter activity; 3.9e-05(36.50x). hydrogen ion transporter activity; 3.9e-05(36.50x). primary active transporter activity; 9.7e-05(27.38x). carrier activity; 4.4e-04(16.85x). cation transporter activity; 5.5e-04(15.10x). | 2 | 113.64 | 0.02930 |
| coenzymes and prosthetic group metabolism; 6.7e-06(25.03x). cofactor metabolism; 6.7e-06(25.03x). coenzyme metabolism; 6.7e-06(25.03x). energy pathways; 7.8e-06(13.04x). monovalent inorganic cation transporter activity; 6.7e-05(31.29x). hydrogen ion transporter activity; 6.7e-05(31.29x). primary active transporter activity; 1.7e-04(23.46x). carrier activity; 7.5e-04(14.44x). cation transporter activity; 9.4e-04(12.95x). | 2 | 97.41 | 0.03920 |
| Developmentally modulated gene cluster. cgc4386\_cluster\_1\_4 | 4 | 13.37 | 0.04800 |
| coenzymes and prosthetic group metabolism; 1.3e-05(21.90x). cofactor metabolism; 1.3e-05(21.90x). coenzyme metabolism; 1.3e-05(21.90x). energy pathways; 2.0e-05(11.41x). monovalent inorganic cation transporter activity; 1.1e-04(27.38x). hydrogen ion transporter activity; 1.1e-04(27.38x). primary active transporter activity; 2.7e-04(20.53x). | 2 | 85.23 | 0.04960 |

### Motifs enriched

|  |  |  |  |  |  |
| --- | --- | --- | --- | --- | --- |
| **Motif** | **Logo** | **Possible orthologs** | **Number of motifs in cluster** | **Enrichment** | **FDR corrected p** |
| ARI3A\_do |  | cfi-1 | 29 | 1.94 | 0.0031 |
| pTH8863 |  | hmg-12 | 29 | 1.92 | 0.0036 |
| pTH9215 |  | C34D1.1 | 31 | 1.79 | 0.0052 |
| POU3F3\_3 |  | ceh-18 (-0.61) | 38 | 1.52 | 0.0064 |
| pTH9916 |  | crh-1 W08E12.1 | 12 | 3.72 | 0.0067 |
| pnr\_SANGER\_5\_FBgn0003117 |  | elt-1 | 36 | 1.53 | 0.0140 |
| pTH3712 |  | B0310.2 D1081.8 | 8 | 5.13 | 0.0140 |
| NR2E3\_f1 |  | nhr-100 (-0.53) | 37 | 1.49 | 0.0160 |
| pTH4425 |  | cfi-1 | 28 | 1.79 | 0.0160 |
| Ets21c\_SANGER\_5\_FBgn0005660 |  | lin-1 | 27 | 1.82 | 0.0170 |
| GM12878\_MEF2C\_HudsonAlpha |  | mef-2 | 35 | 1.54 | 0.0190 |
| ONEC2\_si |  | ceh-48 dsc-1 | 37 | 1.47 | 0.0210 |
| Atf1\_3026 |  | crh-1 | 7 | 5.43 | 0.0220 |
| V$IK1\_01 |  | F26F4.8 | 9 | 4.11 | 0.0230 |
| eve\_FlyReg\_FBgn0000606 |  | ceh-53 hmbx-1 Y116A8C.22 | 16 | 2.51 | 0.0230 |
| FOXF1\_f1 |  | let-381 | 38 | 1.44 | 0.0230 |
| CG2052\_SANGER\_2.5\_FBgn0039905 |  | lin-29 fkh-7 | 41 | 1.35 | 0.0230 |
| pTH9296 |  | C34D1.1 | 32 | 1.61 | 0.0230 |
| rn\_SOLEXA\_5\_FBgn0259172 |  | lin-29 | 40 | 1.38 | 0.0240 |
| MSX2\_f1 |  | ceh-1 | 19 | 2.22 | 0.0250 |
| Sox15\_3457 |  | sox-4 (-0.65) | 29 | 1.70 | 0.0250 |
| pTH9125 |  | egl-13 (-0.6) | 32 | 1.60 | 0.0250 |
| MA0453.1 |  | ceh-18 (-0.61) | 29 | 1.70 | 0.0260 |
| sv\_SOLEXA\_5\_FBgn0005561 |  | pax-2 | 10 | 3.62 | 0.0260 |
| pTH9180 |  | mel-28 | 38 | 1.42 | 0.0300 |
| CREB1\_f1 |  | crh-1 | 21 | 2.03 | 0.0310 |
| MA0049.1 |  | hbl-1 | 34 | 1.52 | 0.0320 |
| Foxj3\_2 |  | lin-31 | 38 | 1.41 | 0.0330 |
| pTH9242 |  | mel-28 | 38 | 1.41 | 0.0330 |
| pTH5828 |  | nhr-84 (-0.59) | 6 | 6.00 | 0.0330 |
| pTH9189 |  | dmd-3 | 36 | 1.45 | 0.0390 |
| tgo\_sima\_SANGER\_5\_FBgn0015014 |  | aha-1 | 8 | 4.15 | 0.0390 |
| V$TCF11\_01 |  | skn-1 | 22 | 1.93 | 0.0400 |
| Foxk1\_1 |  | lin-31 | 20 | 2.04 | 0.0400 |
| V$MYB\_Q6 |  | D1081.8 | 7 | 4.64 | 0.0450 |
| pTH9262 |  | lin-54 | 36 | 1.44 | 0.0450 |
| HOXD8\_1 |  | lin-39 | 32 | 1.53 | 0.0480 |
| V$IK2\_01 |  | F26F4.8 | 5 | 6.83 | 0.0490 |

### Correlated (and anti-correlated) transcription factors

|  |  |
| --- | --- |
| **Transcription factor** | **Correlation** |
| C16A3.4 | 0.67 |
| icd-2 | 0.64 |
| lst-5 | 0.63 |
| Y56A3A.18 | 0.60 |
| C01F6.9 | 0.60 |
| lin-32 | 0.60 |
| mxl-1 | 0.59 |
| hmg-11 | 0.59 |
| Y53F4B.3 | 0.57 |
| zip-7 | 0.57 |
| nhr-64 | 0.56 |
| cebp-2 | 0.53 |
| ceh-13 | 0.53 |
| hmg-6 | 0.52 |
| hlh-15 | 0.51 |
| mxl-2 | 0.50 |
| duxl-1 | 0.49 |
| nhr-273 | 0.49 |
| hmg-5 | 0.48 |
| klf-1 | 0.48 |
| nhr-210 | 0.47 |
| nhr-122 | 0.46 |
| F27D4.4 | 0.46 |
| R144.3 | 0.45 |
| dhhc-1 | 0.45 |
| nhr-214 | -0.66 |
| saeg-2 | -0.66 |
| egrh-1 | -0.66 |
| nhr-17 | -0.66 |
| nhr-158 | -0.67 |
| tag-97 | -0.67 |
| aptf-4 | -0.67 |
| sem-4 | -0.67 |
| crh-2 | -0.68 |
| daf-3 | -0.69 |
| tag-68 | -0.69 |
| nhr-40 | -0.69 |
| dmd-10 | -0.70 |
| zfh-2 | -0.70 |
| ctbp-1 | -0.72 |
| nhr-27 | -0.74 |
| nhr-1 | -0.76 |
| nhr-4 | -0.76 |
| saeg-1 | -0.76 |
| nhr-20 | -0.77 |
| ceh-62 | -0.77 |
| mgl-2 | -0.77 |
| madf-4 | -0.79 |
| F26A10.2 | -0.83 |
| F10B5.3 | -0.85 |

### ChIP peaks enriched

|  |  |  |  |  |
| --- | --- | --- | --- | --- |
| **Gene** | **Experiment** | **Number of upstream peaks** | **Enrichment** | **FDR corrected p** |
| sax-3 | SAX-3\_Larvae-L3-stage | 11 | 3.56 | 0.0073 |
| lsy-2 | LSY-2\_Larvae-L2-stage | 10 | 3.66 | 0.0120 |
| lin-15 | LIN-15B\_Larvae-L4-stage | 9 | 4.03 | 0.0120 |
| ceh-39 | CEH-39\_Embryos | 12 | 3.04 | 0.0140 |
| nhr-77 | NHR-77\_Larvae-L3-stage | 15 | 2.55 | 0.0150 |
| sax-3 | SAX-3\_Larvae-L2-stage | 15 | 2.52 | 0.0170 |
| C16A3.4 | C16A3.4\_Fed-L1-stage-larvae | 16 | 2.29 | 0.0270 |
| lin-35 | LIN-35\_Starved-L1-stage-larvae | 11 | 2.97 | 0.0290 |
| nhr-76 | NHR-76\_Larvae-L3-stage | 12 | 2.76 | 0.0300 |
| F23B12.7 | F23B12.7\_Young-adult | 14 | 2.47 | 0.0310 |
| nhr-77 | NHR-77\_Larvae-L4-stage | 26 | 1.70 | 0.0340 |
| dpl-1 | DPL-1\_Young-adult | 14 | 2.42 | 0.0370 |
| ztf-7 | ZTF-7\_Larvae-L4-stage | 14 | 2.38 | 0.0420 |
| fos-1 | FOS-1\_Larvae-L2-stage | 21 | 1.87 | 0.0430 |
| jun-1 | JUN-1\_Larvae-L1-stage | 14 | 2.37 | 0.0430 |
